# Adapalene inhibits the growth of triple-negative breast cancer cells by S-phase arrest and potentiates the antitumor efficacy of GDC-0941

**DOI:** 10.3389/fphar.2022.958443

**Published:** 2022-08-08

**Authors:** Umar Mehraj, Nissar Ahmad Wani, Abid Hamid, Mustfa Alkhanani, Abdullah Almilaibary, Manzoor Ahmad Mir

**Affiliations:** ^1^ Department of Bioresources, School of Biological Sciences, University of Kashmir, Srinagar, J&K, India; ^2^ Department of Biotechnology, School of Life Sciences, Central University of Kashmir, Ganderbal, J&K, India; ^3^ Biology Department, College of Science, University of Hafr Al Batin, Hafr Al Batin, Saudi Arabia; ^4^ Department of Family and Community Medicine, Albaha University, Albaha, Saudi Arabia

**Keywords:** Breast cancer, triple-negative breast cancer, adapalene, combination therapy, Chou–Talalay, GDC-0941

## Abstract

Although advances in diagnostics and therapeutics have prolonged the survival of triple-negative breast cancer (TNBC) patients, metastasis, therapeutic resistance, and lack of targeted therapies remain the foremost hurdle in the effective management of TNBC. Thus, evaluation of new therapeutic agents and their efficacy in combination therapy is urgently needed. The third-generation retinoid adapalene (ADA) has potent antitumor activity, and using ADA in combination with existing therapeutic regimens may improve the effectiveness and minimize the toxicities and drug resistance. The current study aimed to assess the anticancer efficacy of adapalene as a combination regimen with the PI3K inhibitor (GDC-0941) in TNBC *in vitro* models. The Chou–Talalay’s method evaluated the pharmacodynamic interactions (synergism, antagonism, or additivity) of binary drug combinations. Flow cytometry, Western blotting, and *in silico* studies were used to analyze the mechanism of GDC–ADA synergistic interactions in TNBC cells. The combination of GDC and ADA demonstrated a synergistic effect in inhibiting proliferation, migration, and colony formation of tumor cells. Accumulation of reactive oxygen species upon co-treatment with GDC and ADA promoted apoptosis and enhanced sensitivity to GDC in TNBC cells. The findings indicate that ADA is a promising therapeutic agent in treating advanced BC tumors and enhance sensitivity to GDC in inhibiting tumor growth in TNBC models while reducing therapeutic resistance.

## Graphical Abstract

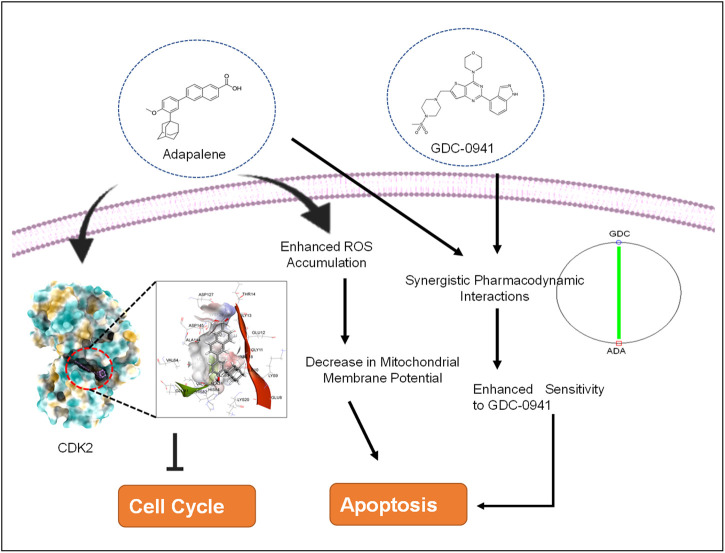

## Introduction

Breast cancer (BC) is one of the most frequent malignancies diagnosed in women worldwide, with the highest incidence and mortality rates (Siegel et al., 2021; Sung et al., 2021). TNBC, an aggressive and invasive subtype of BC, constitutes 20% of all breast malignancies (Hon et al., 2016). TNBC tumors are large, less differentiated, and prone to brain metastasis. Owing to the absence of hormonal receptors (ER and PR) and *HER2* amplification, conventional chemotherapeutic agents continue to be the primary therapeutic approach (Yin et al., 2020). TNBC patients respond favorably to chemotherapy; however, the development of therapeutic resistance limits the prognosis and is associated with poor survival (Yin et al., 2020; Mehraj et al., 2021a; Mehraj et al., 2021b). The need for effective treatment options and treatment strategies, as a result, has become urgent.

Due to the intrinsic instability of tumor cells, which makes therapeutic resistance common, aggressive malignancies such as TNBC cannot be efficiently treated with a single treatment. As a result, combining therapeutic drugs may be more helpful in treating the condition (Lebert et al., 2018; Mir et al., 2020; Yin et al., 2020). In addition, by concurrently targeting different signaling cascades implicated in tumor development in a parallel or linear manner, combination therapy minimizes the chance of chemoresistance and toxicity while retaining or even enhancing the effectiveness of each agent at lower dosages (Lu et al., 2013; Mir et al., 2020). Moreover, combination therapy is a promising technique that can alter the long-term strategy for developing a more effective treatment option for TNBC patients (Mir et al., 2020).

The PI3K/AKT/mTOR signaling cascade is critical for cell biology functions such as metabolism, growth, survival, and genomic stability and has been found aberrant in several malignancies, including BC (Mishra et al., 2021). As a result, inhibitors targeting PI3K/AKT/mTOR signaling are studied extensively (Ellis and Ma, 2019; Mishra et al., 2021). Previously, studies have established that TNBC cells show resistance to GDC-0941, a pan-PI3K inhibitor (Tzeng et al., 2015). Given the lack of targeted medicines for TNBC, modulating current therapy regimens appear to be a potential strategy for developing effective therapies (Ellis and Ma, 2019; Elwakeel et al., 2019; Mehraj et al., 2021c).

Adapalene (ADA), a third-generation retinoid clinically used to treat acne vulgaris on a topical basis, was the second chemical we investigated (Rusu et al., 2020). Numerous research studies on the pharmacological features of ADA have proven its low toxicity and good stability compared to other retinoids. *In vitro* and *in vivo*, it inhibits the proliferation of Hela, CC-531, and HepG2 cells and various cancers (Ocker et al., 2003; Shi et al., 2015; Wang et al., 2019; Ghosalkar et al., 2018; Rusu et al., 2020; Mehraj et al., 2022a). Repurposing ADA for cancer therapy is a promising approach. Herein, we evaluated the therapeutic potential of ADA in TNBC models for improving TNBC cell sensitivity to GDC-0941 (GDC). This is the first study to assess the pharmacodynamic interactions of GDC and ADA in TNBC *in vitro* models.

## Materials and methods

### Chemicals and reagents

Cayman Chemical (Ann Arbor, Michigan 48108, United States) supplied GDC-0941 (Cat. No. 1160) and adapalene (Cat. No. 13655) (Ann Arbor, Michigan 48108, United States). Dulbecco’s modified Eagle medium (DMEM), Roswell Park Memorial Institute medium (RPMI-1640), and fetal bovine serum (FBS) were procured from Gibco, Thermo Fisher Scientific, United States. All the reagents used were of molecular biology or cell culture grade.

### Cell culture

TNBC cell lines (MDA-MB-231 and MDA-MB-468) and ER + cell line MCF-7 were procured from the Cell Repository, National Centre for Cell Science (NCCS) Pune, India. Prof. Annapoorni Rangarajan (IISC, India) graciously provided the murine TNBC cell line 4T1. MDA-MB-231, MCF-7, and MDA-MB-468 cells were cultured in DMEM with 10% FBS and 1% penicillin–streptomycin. The murine TNBC cell line, 4T1, was cultured in RPMI-1640 with FBS (10%) and penicillin–streptomycin (1%). The BC cell lines were maintained at 37°C in a humidified CO_2_ incubator (5%).

### Single-drug cytotoxicity assay

Cell viability assay was used to assess the potency of ADA and GDC and to generate a dose–response curve required for the Chou–Talalay model for designing binary drug combinations (Chou, 2006; Zhang et al., 2016). In 96-well plates, BC cells (MDA-MB-468, 4T1, MDA-MB-231, and MCF-7) were cultured at 3 × 10^3^ cells/well. Seven distinct concentrations of GDC, ADA, or drug vehicle (DMSO), each with four replicates, were given the next day. After 72 h of incubation, the drug solutions were replaced with a fresh media containing 5 mg/ml MTT (Invitrogen), and the growth inhibition was evaluated using the following equation (Eq. 1):

$$\text{\% Inhibition}=\left[1-\left(\frac{\text{OD treated Cells}}{\text{OD vehicle control Cells}}\right)\right]\times 100,$$ (1)

where “OD-treated cells” defines the mean absorbance of cells incubated with therapeutics, “OD vehicle control” implies the mean absorbance of cells treated with a complete cell culture medium containing 0.1% DMSO.

### Constant ratio cytotoxicity test for binary drug combinations

The cytotoxicity assay of single drugs in BC models laid the groundwork for the combined evaluation of GDC and ADA. Six distinct equipotent GDC–ADA combinations were constructed using the IC_50s_ values of the two drugs and evaluated in four repetitions in different cell lines. As Chou and Talalay recommended, the equipotent constant ratio method was used for all combinations. In this method, the amount of each agent in the combination is the same (Chou, 2006; 2010). Following a 72-hr treatment period, the cytotoxic effects of drugs as individual agents or in combination were assessed. As indicated in Eq. 1, the percentage inhibition for each combination was calculated.

### Adoption of the Chou–Talalay approach for calculating the combination index and DRI

The combination index (CI) value—a dimensionless variable used to identify and quantify pharmacological interaction—was generated for binary combinations using CompuSyn software application, implementing the combination index equation (Eq. 2). When the CI value equals one, an additive impact is obtained. Synergistic interaction is observed when CI < 1 and antagonistic interaction when CI > 1.

$${\left(\text{CI}\right)}^{2}=\frac{{\left(\text{D}\right)}_{1}}{{\left(\text{Dy}\right)}_{1}}+\frac{{\left(\text{D}\right)}_{2}}{{\left(\text{Dy}\right)}_{2}}\;=\frac{{\left(\text{D}\right)}_{1}}{{\left(\text{Dm}\right)}_{1\;}{\left[\text{fa }/\left(1-\text{fa}\right)\right]}^{1/\text{m}1}}\;+\;\frac{{\left(\text{D}\right)}_{2}}{\left({\text{Dm}}_{2}\right){\left[\text{fa}/\left(1-\text{fa}\right)\right]}^{1/\text{m}2}}\;,$$ (2)

where (Dx)1 is the concentration of drug 1 that alone reduces cell viability by x percent, (Dx)2 is the drug 2 concentration that alone reduces cell viability by x percent, and (D)1 and (D)2 are the concentrations of drug 1 (D1) and drug 2 (D2) taken together that reduce cell viability by x percent. The values of (Dx)1 and (Dx)2 can be easily obtained by rearrangement of the median-effect equation (Sung et al., 2021) as follow.

$$D=Dm\;{\left[\frac{fa}{1-fa}\right]}^{1/m}.$$

The dimensionless function, dose reduction index or DRI, evaluates and indicates the magnitude, by which the concentration of the individual agent in a drug combination may be lowered compared to the doses of each drug alone at a given fractional inhibition. It was generated automatically by the CompuSyn program for experimental drug combinations based on the DRI equations (Chou, 2006) as follows:

$${\left(\text{DRI}\right)}_{1}=\frac{{\left(\text{Dx}\right)}_{1}}{\text{D}1}\;,{\left(\text{DRI}\right)}_{2}=\;\frac{{\left(\text{Dx}\right)}_{2}}{\text{D}2},\;{\left(\text{DRI}\right)}_{3}=\;\frac{{\left(\text{Dx}\right)}_{3}}{\text{D}3}\ldots \text{ etc}.$$

DRI greater than one implies a desirable dosage decrease, DRI less than one suggests a detrimental dose reduction, and DRI equal to one indicates zero dose reduction (Chou, 2006).

### Proliferation assay

After evaluating pharmacodynamic interactions, we examined the effect of the synergistic drug combination of GDC and ADA on cell proliferation time-dependently. In a 96-well plate, the cells were seeded (3 × 10^3^ cells/well) and treated with ADA or GDC alone or in combination at concentrations below the IC_50_ values. After 24–72 h of incubation, according to the manufacturer’s instructions, the proliferation of cells was determined using the Vybrant Proliferation Kit (Thermo Fisher Scientific, United States). GraphPad Prism 8.4.3 and a two-way ANOVA were employed for statistical analysis, followed by a Tukey test for multiple comparisons.

### Colony formation assay

To assess the impact of ADA, GDC, and their combined effect on the colony formation of cells, cells were seeded in six-well plates at a density of 1,000–1,500 cells per well (Elbaz et al., 2015). After 48 h, the media was replaced with a fresh medium, supplemented with therapeutics. The assay was performed for 14–18 days. The medium containing therapeutics was replenished every 3 days, and colonies were observed in the wells using an inverted microscope. Once substantial colonies were formed, they were fixed with 3.7 percent paraformaldehyde (in PBS) and stained with crystal violet (0.05%). The plates were photographed, and the colonies were counted using the ImageJ program. Each cell type and treatment combination was subjected to the experiment thrice.

### Wound healing assay

We utilized the Wound Healing Assay Kit (Cell Biolabs, Inc. United States) to investigate the impact of GDC and ADA and their combination on the migration of the highly invasive TNBC cell lines MDA-MB-231 and 4T1. On a 24-well plate, cells were seeded at 70% confluency and allowed to attach overnight with implanted scratch inserts. After 24 h, the scratch inserts were gently removed, and the cells were rinsed with PBS. Fresh media with therapeutics was added, and cell migration was assessed after 48 h of treatment. The cells were fixed in 3.7% paraformaldehyde (in PBS) and stained with Giemsa stain (in PBS). The cells were photographed, and the migration of cells into the wound region was analyzed and quantified using ImageJ software (Pijuan et al., 2019).

### Mammosphere formation assay

MDA-MB-231 cells (1 × 10^4^) were seeded as a single-cell suspension in 2-ml DMEM/F12 (Gibco, 11320033), supplemented with 1x B27 supplement (Invitrogen, 17504044) and SingleQuot™ (Lonza, CC-4136) into each well of ultralow attachment 6-well plates (Corning, 3471) (Klopp et al., 2010). The next day, the cells were treated with therapeutics as single agents or in combination, and the cells were cultured for 5–10 days later with media added every 3 days. Spheres were imaged under an inverted phase-contrast microscope (Nikon).

### Reactive oxygen species measurement assay

MDA-MB-231 cells were grown in 24-well culture plates and treated with ADA, GDC, or both for 24 h. Next, the cells were stained with 10-μM 2′,7′-dichlorofluorescin diacetate (DCFDA) (Sigma-Aldrich) for 30 min in the dark, and fluorescence intensity was measured using a fluorometer.

### Mitochondrial membrane potential analysis

Rhodamine 123 (Rh 123) staining was used to assess changes in mitochondrial membrane potential. The transition of mitochondria from a polarized to a depolarized state during the induction of apoptosis results in leakage of the dye, consequently resulting in a decrease in Rh 123 fluorescence intensity. The cells were grown in 24-well plates and treated with GDC, ADA, or both for 24 h. The cells were collected and incubated with 10-μM Rh 123 for 15 min at 37°C in the dark. Next, the cells were resuspended in PBS and analyzed immediately using an Agilent fluorescence spectrophotometer.

### Annexin V assay

To investigate the mechanism behind the antitumor activity of ADA, GDC, and their combination, we utilized a BD Biosciences Annexin V apoptosis detection kit. MDA-MB-231 was treated with GDC, ADA, or both for 24 and 48 h. All the cells were collected, including free-floating and adherent cells, and stained with the fluorescent dyes FITC-Annexin V and 7-AAD as recommended by the manufacturer. Flow cytometry was performed at the Department of Biotechnology, National Institute of Technology, Rourkela, Odisha, India, on a BD Accuri™ C6 flow cytometer (Mehraj et al., 2022b).

### Molecular docking

To further investigate the molecular target of ADA in breast tumor cells, we utilized the molecular docking technique to validate the targets. Previous studies have demonstrated that ADA selectively targets CDK2 in cancer cells. As CDK2 is highly upregulated in BC patients, targeting CDK2 in combination with conventional therapy is a promising approach. Autodock v 4.2.6 was used to perform docking investigations of ADA and CDK2. The predetermined co-crystallized X-ray structure of CDK2 (5NEV) from the RCSB PDB was used to calculate the binding cavity of proteins. The co-crystallized ligand was used to compute the residue locations within the 4-Å radius. As part of the cavity selection process, chimera (https://www.cgl.ucsf.edu/chimera/) was used to remove co-crystallized ligands, and then the energy was minimized using the steepest descent and conjugate gradient algorithms. Both receptor and target compound were then saved in pdbqt format after combining non-polar hydrogens. Molecular docking was performed within a grid box dimension 14 × 14 × 13 Å. It was necessary to design grid boxes with particular dimensions and 0.3 Å spacing. Docking experiments of the protein–ligand complex were carried out following the Lamarckian genetic algorithm (LGA). There were three replicates of molecular docking investigations, each of which included 50 solutions, a population size of 500, 2,500,000 evaluations, a maximum generational number of 27, and all other parameters were left at their default values. Once the docking was complete, the RMSD clustering maps were generated by re-clustering with the clustering tolerances of 0.25, 0.50, and 1 to find the best cluster with the lowest energy score and the most populations.

### Molecular dynamics simulation

The Desmond 2020.1 from Schrödinger, LLC was used to run MD simulations on dock complexes for CDK2 with ADA. SPC water molecules and the OPLS-2005 force field were utilized in this system (Jorgensen et al., 1983) in a period boundary salvation box of 10 × 10 × 10 Å dimensions. Na^+^ ions were supplied to the system to neutralize the charge, and 0.15 M of NaCl solution was added to replicate the physiological environment. When retraining with the complex CDK2-ADA, the system was first equilibrated using an NVT ensemble for 100 ns. After the preceding phase, a 12-ns NPT ensemble run was used to perform a quick equilibration and reduction. The NPT ensemble was set up using the Nose–Hoover chain coupling approach (Li et al., 2003) and run at 27°C for 1.0 ps under a pressure of 1 bar throughout the study. A time step of 2 fs was employed in this experiment. With a relaxation duration of 2 ps, the Martyna–Tuckerman–Klein barostat method was utilized for pressure control. Ewald’s particle mesh approach was used to calculate long-range electrostatic interactions; the radius for coulomb interactions was fixed at 9 nm. The bonded forces were calculated using the RESPA integrator with a time step of 2 fs for each trajectory. Calculations were made to track the stability of MD simulations using parameters such as the root mean square deviation (RMSD), gyroradius, root mean square fluctuation (RMSF), number of hydrogen atoms (H-bonds), and solvent accessible surface area (SASA).

### Western blotting

Cells were grown in 6-cm dishes and treated with ADA for 24 h. After the drug treatment, the cells were lysed with lysis buffer NP40 (Invitrogen, Thermo Fisher Scientific), supplemented with protease and phosphatase inhibitors. Next, protein concentrations were determined using the BCA assay kit (Pierce™ BCA Protein Assay Kit, Cat No. 23227, Thermo Scientific). Equal amounts of protein were separated by electrophoresis on SDS-polyacrylamide gels and electroblotted onto polyvinylidene difluoride membranes. BSA (5%) was used to block non-specific binding for 1 h at room temperature. Protein bands were probed using specific primary antibodies, viz., CDK2 (78B2) rabbit mAb (CST, Cat No. 2546, dilution-1: 1000) and horseradish peroxidase-conjugated secondary antibodies and visualized using an ECL kit (Bio-Rad, Hercules, CA). ImageJ software analyzed the intensity of immunoreactive protein bands and normalized them with GAPDH (CST, Cat No 2118, dilution-1:1000) as the loading control.

### Cell cycle analysis

MDA-MB-231 cells were seeded in 12-well plates at 50% confluency and allowed to adhere overnight, followed by serum starvation for cell cycle synchronization. Next, the cells were treated for 24 and 48 h with ADA, GDC, and a combination of ADA and GDC. After treatment, the cells were trypsinized and fixed in 75% ethanol. After washing, the cells were stained with a solution containing PI (0.5 mg/ml) and RNase A (10 mg/ml). The cells were filtered prior to flow cytometry, using a 70-m cell strainer. Flow cytometry was performed at the NIT Rourkela, India (Mehraj et al., 2022b).

### Statistics

IC_50s_ values were calculated using non-linear regression analysis in GraphPad Prism. The statistical significance was analyzed using the one-way or two-way ANOVA in GraphPad Prism V 8.43, followed by Tukey multiple comparisons test. *p* < 0.05 was considered significant.

## Results

### Cytotoxicity assay for each single drug

**FIGURE 1:**
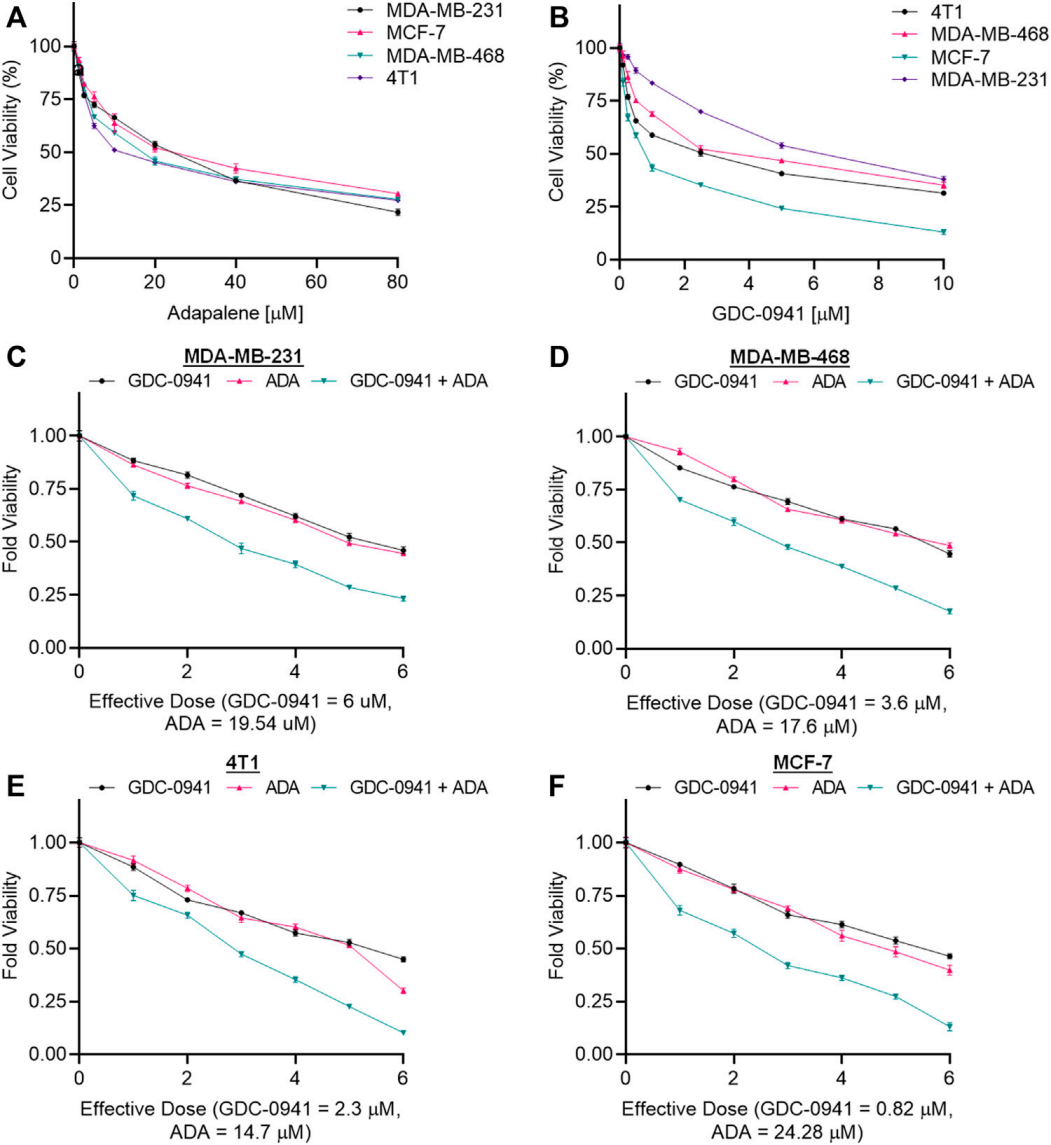
Adapalene and GDC reduce TNBC cell growth *in vitro*. Single-drug cytotoxicity assays of **(A)**. adapalene and **(B)** GDC-0941 in TNBC cell lines and MCF-7 cells. Both ADA and GDC inhibited tumor growth in a dose-dependent manner. The IC_50_ values were calculated using GrpahPad Prism V8.4.3. Combination treatment shows an enhanced reduction in cell growth in **(C)**. MDA-MB-231 **(D)**. MDA-MB-468 **(E)**. 4T1 and **(F)** MCF-7 cells. Enhanced reduction in cell viability was observed upon combination treatment with ADA and GDC, indicating positive pharmacodynamic interactions.

The cell viability assay using MTT reagent was carried out to evaluate the cytotoxicity of GDC and ADA alone against BC cell lines, and GraphPad Prism 8 was used to generate dose–effect curves and obtain IC_50_ values for single drugs (Figures 1A,B). GDC and ADA were both cytotoxic to all breast cancer cell lines dose-dependently. The IC_50_ values of GDC in MDA-MB-231, MCF-7, MDA-MB-468, and 4T1 were 6.0, 0.82, 3.6, and 2.3 µM, respectively. GDC-0941 demonstrated high cytotoxicity in ER + MCF-7 cells, while TNBC cells showed resistance to GDC, with MDA-MB-231 showing the highest resistance. ADA showed an IC_50_ of 17.57, 19.54, 24.28, and 14.7 µM in MDA-MB-468, MDA-MB-231, MCF-7, and 4T1, respectively. ADA demonstrated high cytotoxicity in murine TNBC cells (4T1). The ER + cell line was responsive to ADA, while TNBC showed high sensitivity toward ADA.

### Cytotoxicity assay of binary drug combination

**FIGURE 2:**
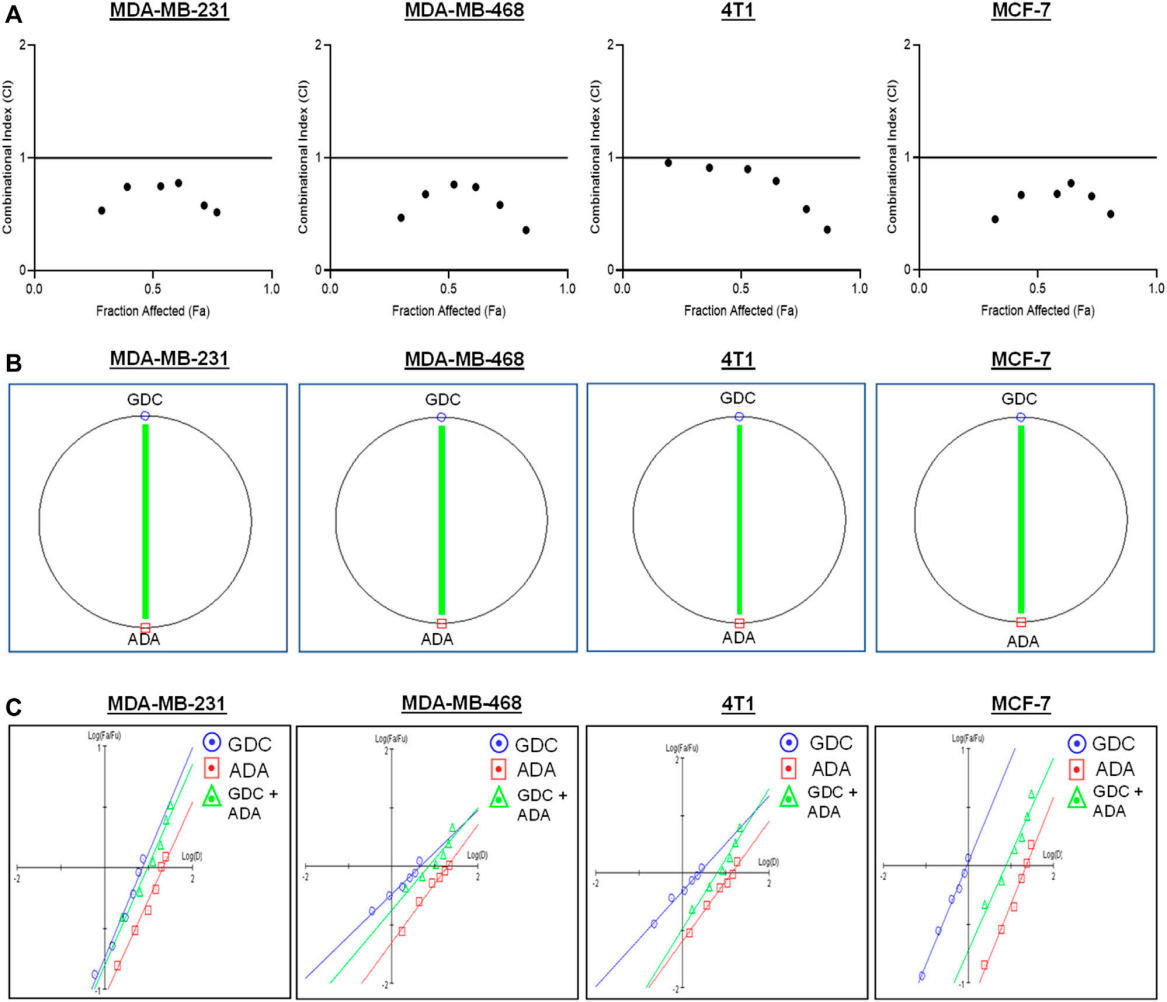
Adapalene and GDC show synergistic pharmacodynamic interactions in inhibiting the growth of TNBC cells **(A)**. Combination Index (CI) plots of MDA-MB-231, MDA-MB-468, 4T1, and MCF-7 cells. The CI plots showed significant synergism between ADA and GDC in TNBC cells **(B)**. Polygonograms of MDA-MB-231, MDA-MB-468, 4T1, and MCF-7 cells **(C)**. Median Plots of MDA-MB-231, MDA-MB-468, 4T1, and MCF-7 cells.

The conditions of the Chou–Talalay method were met by the results of the single-drug cytotoxicity experiment, so the *in vitro* pharmacodynamic drug interaction study could begin. We analyzed a constant ratio combination design to examine all potential binary drug combinations. After 72 h of treatment, cell viability was evaluated Figures 1C–F. The combination of GDC and ADA showed an enhanced reduction in cell viability of BC cells at very low doses, demonstrating positive drug–drug interactions of GDC and ADA in BC cell lines. CompuSyn software was further utilized to calculate the CI, DRI values, and dose–inhibition curve parameters (Table 1). For MCF-7, MDA-MB-468, and MDA-MB-231, a flat sigmoidal (m < 1) curve was observed with an r-value (linear correlation coefficient) of approximately 0.95. 4T1 cells had a sigmoidal curve (m > 1) with approximately 0.95 for *r*. Also, the CompuSyn-calculated CI values could achieve synergistic interactions as demonstrated with the CI less than one at precise combinations (Table 1; Figure 2A). The median-effect blots of all tested drug combinations are shown in Figure 2C.

**TABLE 1:** Experimental design and data summary of the dose–effect curve and Chou–Talalay parameters of GDC-0941 and adapalene drug combinations against breast cancer cell lines after 72 h treatment period.

Cell line	GDC-0941 (G) (µM)	Adapalene (A) (µM)	Fraction affected (Fa)	Parameters					
				m	Dm	r	CI	DRI	
MDA-MB-231	0.1 * IC_50_	0.1 * IC_50_	0.28	0.83	9.4	0.98	0.53	G = 4.15	A = 3.42
	0.25 * IC_50_	0.25 * IC_50_	0.39				0.74	G = 2.90	A = 2.50
	0.5 * IC_50_	0.5 * IC_50_	0.53				0.75	G = 2.80	A = 2.54
	0.75 * IC_50_	0.75 * IC_50_	0.60				0.77	G = 2.66	A = 2.49
	**IC**_**50**_ **(6)**	**IC**_**50**_ **(19.5)**	0.71				0.57	G = 3.49	A = 3.42
	1.25 * IC_50_	1.25 * IC_50_	0.76				0.51	G = 3.85	A = 3.87
MDA-MB-468	0.1 * IC_50_	0.1 * IC_50_	0.29	0.88	7.2	0.95	0.46	G = 3.87	A = 4.77
	0.25 * IC_50_	0.25 * IC_50_	0.40				0.67	G = 2.91	A = 2.99
	0.5 * IC_50_	0.5 * IC_50_	0.52				0.76	G = 2.86	A = 2.41
	0.75 * IC_50_	0.75 * IC_50_	0.61				0.73	G = 3.21	A = 2.33
	**IC**_**50**_ **(3.6)**	**IC**_**50**_ **(17.6)**	0.71				0.58	G = 4.56	A = 2.75
	1.25 * IC_50_	1.25 * IC_50_	0.82				0.35	G = 8.75	A = 4.10
4T1	0.1 * IC_50_	0.1 * IC_50_	0.24	1.22	6.3	0.98	0.95	G = 1.86	A = 2.39
	0.25 * IC_50_	0.25 * IC_50_	0.34				0.91	G = 2.16	A = 2.22
	0.5 * IC_50_	0.5 * IC_50_	0.52				0.89	G = 2.38	A = 2.08
	0.75 * IC_50_	0.75 * IC_50_	0.64				0.79	G = 2.89	A = 2.23
	**IC**_**50**_ **(2.3)**	**IC**_**50**_ **(14.7)**	0.77				0.54	G = 4.63	A = 3.06
	1.25 * IC_50_	1.25 * IC_50_	0.89				0.36	G = 7.65	A = 4.35
MCF-7	0.1 * IC_50_	0.1 * IC_50_	0.31	0.82	7.58	0.98	0.45	G = 4.77	A = 4.13
	0.25 * IC_50_	0.25 * IC_50_	0.42				0.66	G = 3.27	A = 2.77
	0.5 * IC_50_	0.5 * IC_50_	0.57				0.67	G = 3.26	A = 2.69
	0.75 * IC_50_	0.75 * IC_50_	0.63				0.77	G = 2.88	A = 2.35
	**IC**_**50**_ **(0.82)**	**IC**_**50**_ **(24.2)**	0.72				0.65	G = 3.42	A = 2.74
	1.25 * IC_50_	1.25 * IC_50_	0.86				0.49	G = 4.57	A = 3.59

m, Median; Dm, IC50; r, linear correlation coefficient CI, Combinational Index.

### CompuSyn software’s computer simulation

Utilizing the median effect and the combination index equations and the automation capabilities of the CompuSyn program, an algorithm was developed to simulate the computed CI and DRI values at various fraction affected (Fa) levels other than actual dosage. The simulated CI at different Fa levels was significantly synergistic, further validating *in vitro* results. The program generated the simulated Fa-Log CI plot, Fa-DRI plot, and isobolograms for each drug combination (Supplementary Figures S1–S4). The simulated CI and DRI values at 50, 75, 90, and 95% fraction affected are shown in Table 2. Apart from that, polygonograms of drug combinations at 50% Fa levels were designed to provide a visual comparison of the kind and magnitude of drug interactions (Figure 2B). The continuous line represents synergistic interaction, whereas the dashed line represents antagonistic interaction. The width of the line indicates the level of synergy or competition. Based on the simulated CI and DRI, it was confirmed that all tested combinations demonstrated synergistic interactions of various inhibitory magnitudes, demonstrating that ADA and GDC interact synergistically.

**TABLE 2:** Summary of CompuSyn simulated CI and DRI values for GDC-0941 and adapalene combination in breast cancer cell lines at 50, 75, 90, and 95% growth inhibition.

Cell line	Drug combination GDC (G) + ADA (A)	CI values at inhibition of				DRI values at inhibition of			
		50%	75%	90%	95%	50%	75%	90%	95%
MDA-MB-231	G + A	0.63	0.64	0.64	0.65	G = 2.20	G = 8.25	G = 30.92	G = 75.91
						A = 7.18	A = 26.89	A = 100.71	A = 247.21
MDA-MB-468	G + A	0.57	0.56	0.58	0.61	G = 1.23	G = 4.31	G = 15.09	G = 35.39
						A = 6.01	A = 21.07	A = 73.78	A = 173.02
4T1	G + A	0.75	0.57	0.44	0.37	G = 0.85	G = 2.10	G = 5.17	G = 9.52
						A = 5.49	A = 13.46	A = 33.05	A = 60.87
MCF-7	G + A	0.59	0.66	0.73	0.79	G = 0.24	G = 0.94	G = 3.58	G = 8.89
						A = 7.34	A = 27.91	A = 106.14	A = 263.29

### Adapalene enhances sensitivity to GDC-0941 in triple-negative breast cancer cells

**FIGURE 3:**
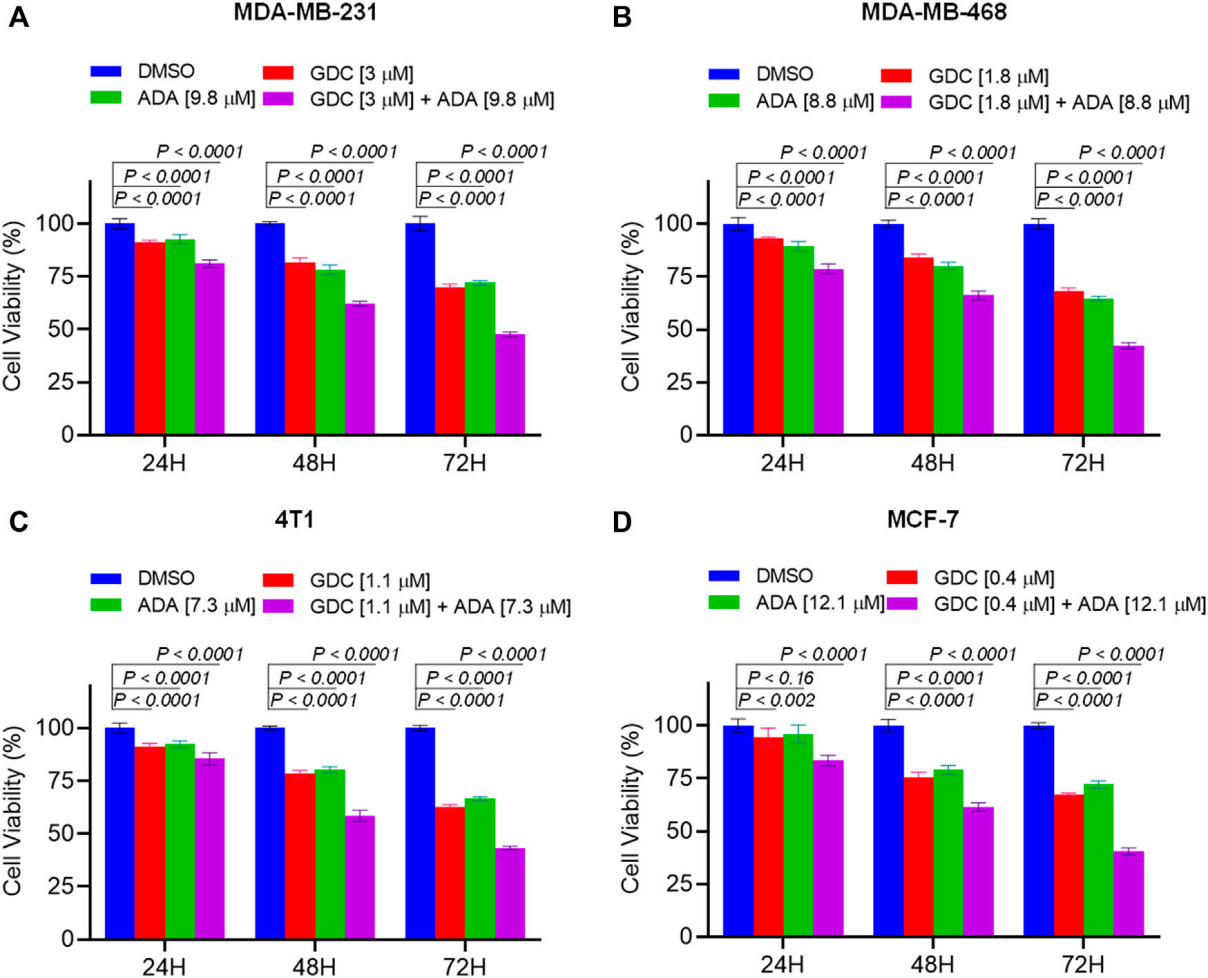
Combination of GDC-0941 and adapalene reduces tumor cell proliferation. The combination of GDC and ADA inhibited proliferation of **(A)**. MDA-MB-231 **(B)**. MDA-MB-468 **(C)**. 4T1 **(D)**. MCF-7 in a synergistic manner. Data are mean ± SD. *p*-values were determined by two-way ANOVA followed by Tukey’s multiple comparisons test. Significant reduction in cell viability was observed when treated in a time-dependent manner with combined treatment of GDC and ADA showing a maximal effect.

We further evaluated the synergetic drug combination of GDC and ADA in a time-dependent manner. We proceeded with a single synergistic drug combination below individual IC_50_ value among several drug combinations designed earlier. The cell viability was analyzed at 24, 48, and 72 h using the Vybrant cell proliferation kit (Invitrogen, Thermo Fisher, United States), following the manufacturer’s protocol. Combinatorial treatment significantly reduced cell viability compared to single-agent treatment (Figure 3). The results demonstrate that GDC and ADA in combination enhance the antiproliferative effect of each other synergistically. Moreover, the sensitivity of TNBC cells toward GDC significantly increased upon co-treatment with ADA. The trend was seen in all three time periods.

### Combination of GDC-0941 and adapalene reduces colony formation and migration of triple-negative breast cancer cells

**FIGURE 4:**
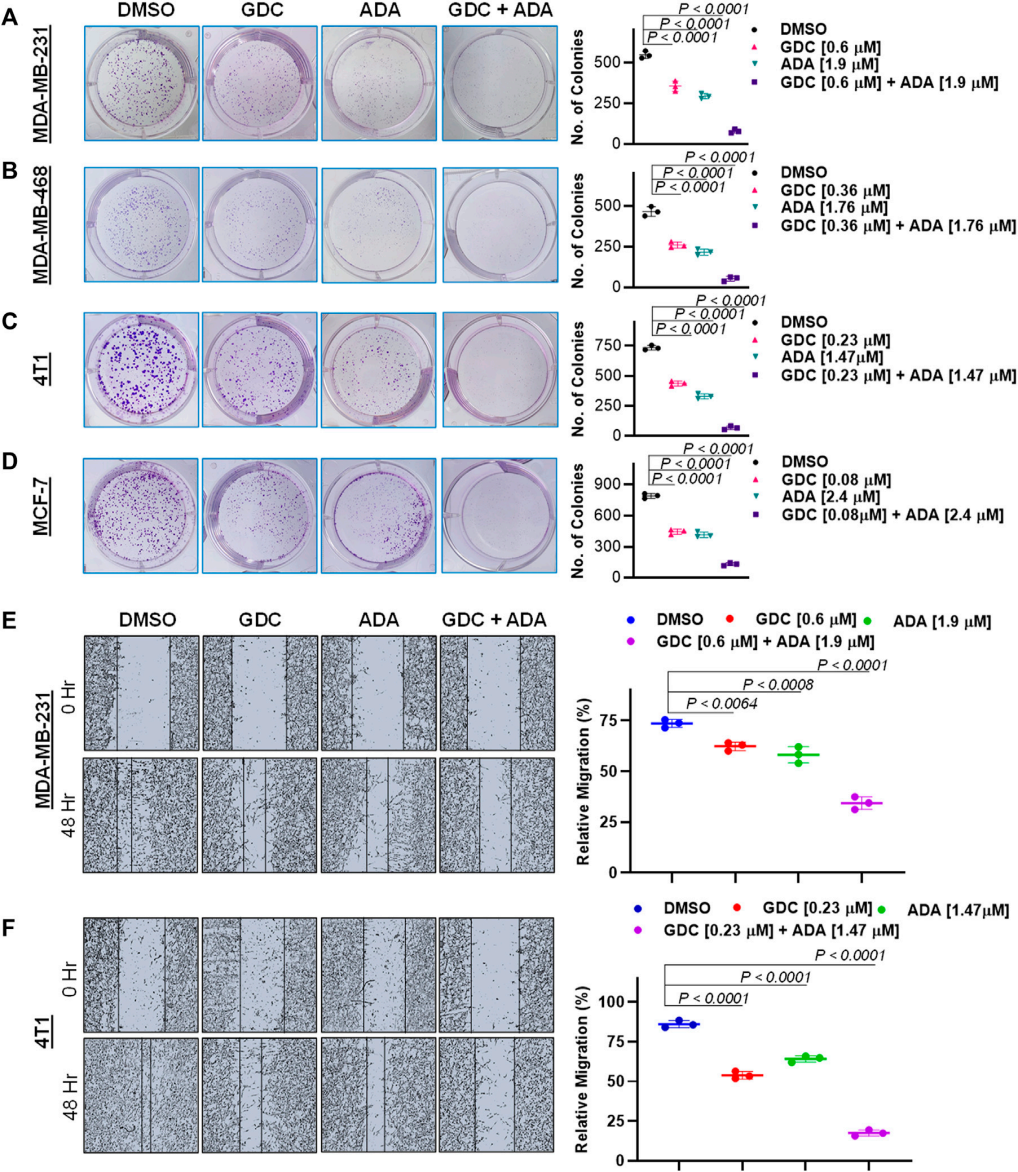
Combination of GDC-0941 and adapalene reduces colony formation and migration potential of TNBC cells. Representative images and quantification of colony formation assay data of **(A)**. MDA-MB-231, **(B)**. MDA-MB-468, **(C)**. 4T1 and, **(D)**. MCF-7 cells are treated with drug vehicle (DMSO), GDC, ADA, or a combination of GDC & ADA. The right panels show the quantification of colonies formed under each treatment condition described in the left panels. Data are mean ± SD. P values were determined by one-way ANOVA followed by Tukey's multiple comparisons test. Representative images and quantification of migration assay data of **(E)**. MDA-MB-231, **(F)**. 4T1. The right panels show the relative migration under control, single treatment and combination of GDC and ADA described in the left panels. Data are mean ± SD. *p*-values were determined by one-way ANOVA followed by Tukey's multiple comparisons test. Data are representative of at least three independent experiments.

Experiments with colony formation in BC cell lines were utilized to confirm and assess the synergistic interactions of ADA and GDC. While treatment with GDC and ADA alone decreased colony formation, treatment with the combination of GDC and ADA resulted in an enhanced decrease in colony formation compared to individual drug treatments. Also, the number of colonies in each treated cell line was almost equivalent when treated alone; however, the number of colonies was significantly reduced when treated in combination. The study demonstrated that ADA as a single agent reduces tumor cell growth, inhibiting the colony formation of breast tumor cells (Figures 4A–D).

To colonize distant organs, cancer cells must penetrate the ECM and undergo the multistep phenomena of metastasis (Neophytou et al., 2018). As a result, inhibiting cell migration may be vital for limiting metastasis. This investigation sought to examine the effect of the GDC–ADA combination on the motility of tumor cells. CytoSelect^TM^ 24-Well Wound Healing Experiment Kit was used to perform the assay in 24-well plates. MDA-MB-231 and 4T1 cells were treated for 48 h with GDC and ADA alone or in combination, and cell movement was assessed using ImageJ software. ADA showed a high reduction in migration of MDA-MB-231 and 4T1 cells. The combination of GDC and ADA significantly reduced migration compared to control cells or cells treated with GDC or ADA alone (Figures 4E,F).

**FIGURE 5:**
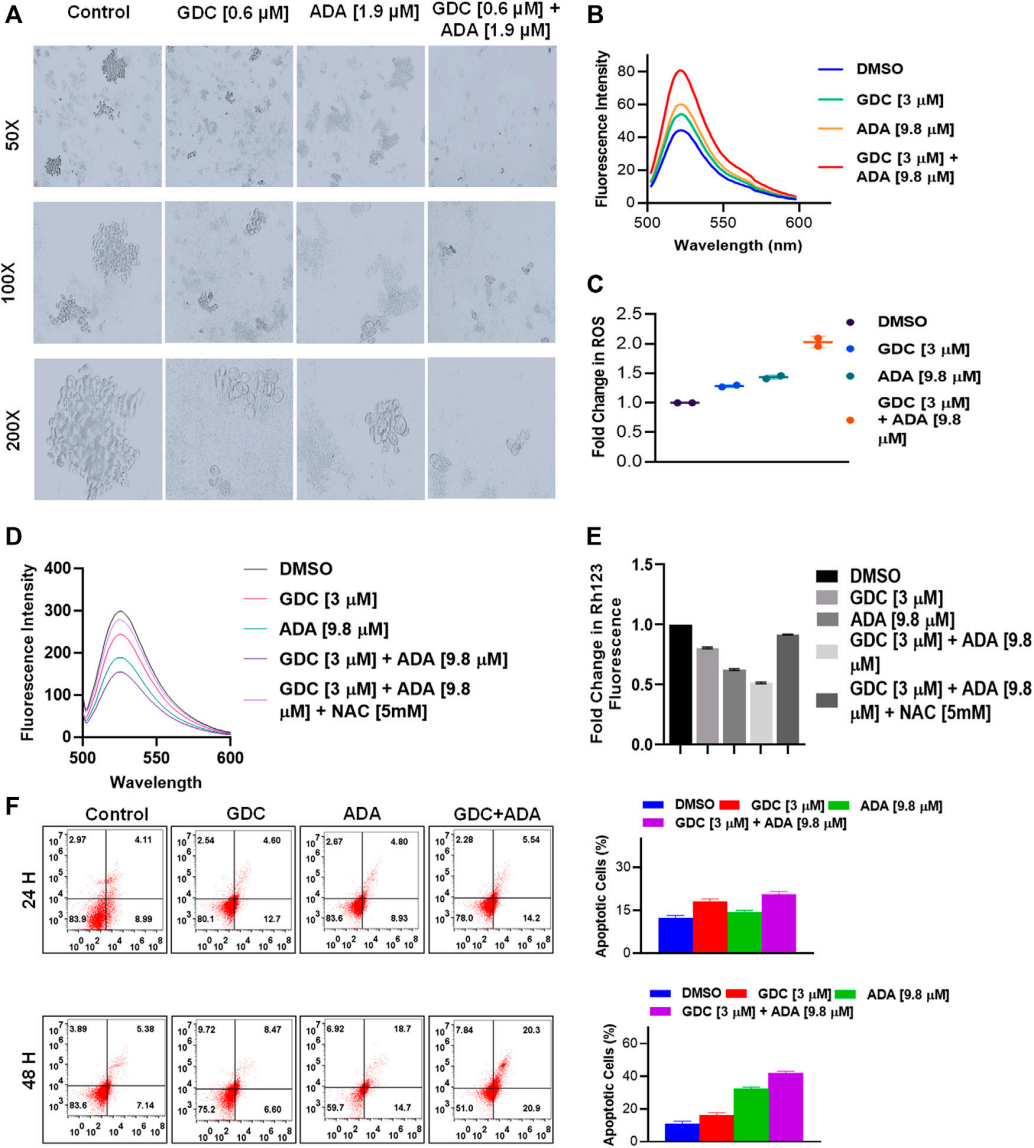
GDC-0941 and adapalene affect the anchorage-independent growth of MDA-MB-231 cells and enhance apoptosis. **(A)**. Representative images of the spheroid assay. Treatment with the combination of GDC and ADA significantly reduced the growth of TNBC cells in ultra-low attachment plates. **(B)**. Fluorescence intensity and **(C)**. fold change in ROS levels in MDA-MB-231 cells treated with ADA (9.8 μM) and GDC (3 μM) alone or in combination for 24 hrs. **(D)**. fluorescence intensity and **(E)**. fold change in Rh123 staining levels in MDA-MB-231 cells treated with ADA or GDC alone or in combination both showing decrease in mitochondrial membrane potential upon treatment. **(F)**. Annexin V & &-AAD staining showed increased apoptotic cells in plates treated with the combination of GDC and ADA after 24 h or 48 hr periods.

Also, GDC and ADA, in combination, significantly repressed the anchorage-independent growth of MDA-MB-231 cells (Figure 5A) and suppressed mammosphere formation. These results further support that GDC and ADA have significant tumor-reducing activity in TNBC.

### Combined treatment with GDC-0941 and adapalene disrupts mitochondrial membrane potential and enhances reactive oxygen species production, triggering apoptosis of triple-negative breast cancer cells

Next, we set out to investigate the mechanisms underlying the anticancer activity of ADA and the synergistic effect of GDC and ADA. Therefore, we measured the intracellular ROS levels after ADA and GDC co-treatment and individual treatment. The results indicated that the combined treatment significantly increased ROS levels in MDA-MB-231. In addition, we found that the treatment with ADA and GDC alone induced ROS generation. However, ROS levels were higher in ADA treated, but the combined treatment resulted in a two-fold increase in ROS levels (Figures 5B,C). ROS generation is associated with mitochondrial membrane potential (MMP) disruption, a critical event in apoptosis initiation, which can be measured by Rh 123 staining. We found that the percentage of cells treated with ADA, GDC, or both showed low Rh 123 fluorescence intensities compared to untreated controls. Moreover, the membrane disruption decreased upon exposure to NAC for 2 h before co-treatment of GDC and ADA, further validating the involvement of ROS in the synergistic interaction of GDC–ADA. These results suggest that oxidative injury, resulting in disruption of mitochondrial membrane potential, may significantly enhance lethality induced by the combined treatment of MDA-MB-231 cells with ADA and GDC (Figures 5D,E).

Furthermore, we utilized Annexin V and 7-AAD staining to assess the apoptosis induction potential of ADA/GDC or a combination of both. MDA-MB-231 cells were treated for 24 and 48 h, followed by staining with Annexin V and 7-AAD. Flow cytometry analysis revealed that ADA induction tumor cell death *via* induction of apoptosis and apoptosis enhanced significantly upon combination treatment (Figure 5D). GDC also showed apoptosis, but apoptosis in combination treatment was highly significant compared to single-agent treatments.

### Molecular docking showed high binding of adapalene with CDK2

**FIGURE 6:**
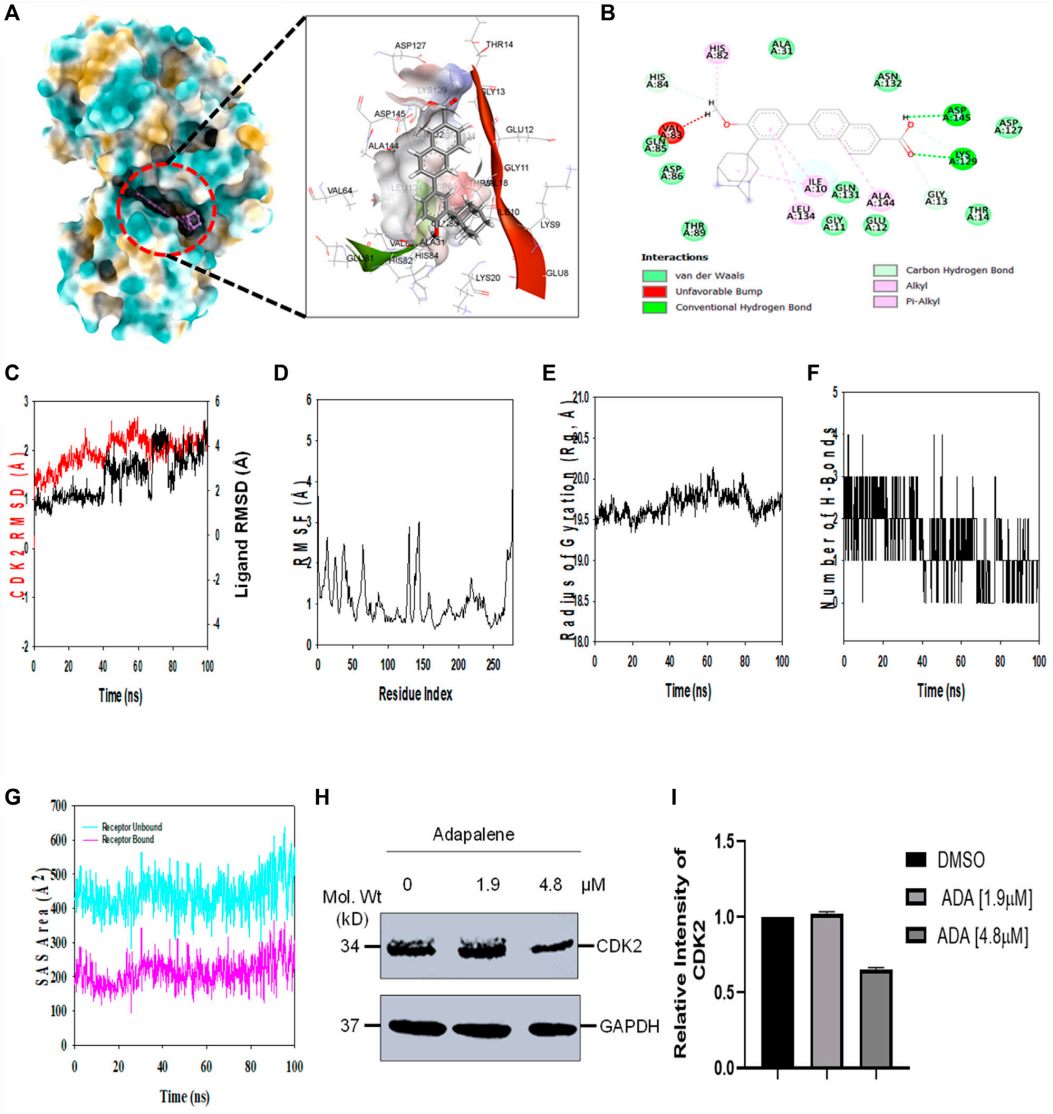
ADA shows high binding affinity and stability with CDK2 **(A)**. Analysis of the binding pose of ADA at the binding cavity of CDK2 on the left panel and 3D cavity interaction of residues with ADA **(B)**. 2D interaction of ADA showing various interactions at the binding cavity of CDK2 **(C)**. RMSD plot displaying the molecular vibration of Cα backbone of CDK2 (red) and ADA (black) **(D)**. RMSF plot showing the fluctuations of respective amino acids throughout the simulation time 100 ns for CDK2-ADA **(E)**. The radius of the gyration plot to deduce the compactness of CDK2 bound to ADA **(F)**. The number of hydrogen bonds formed between CDK2-ADA during the 100 ns simulation time scale **(G)**. Solvent accessible surface area (SAS Area) displays the unbound area at the binding pocket (cyan) and bound ADA with CDK2 **(H)**. Immunoblot of CDK2 upon treatment with ADA. ADA reduced CDK2 in a dose-dependent manner **(I)**. Relative intensity of CDK2 protein levels upon treatment with ADA.

Molecular docking studies were performed to decipher the binding aspects of CDK2 with ADA. The images of docked complexes, molecular surfaces, and 2D and 3D interactive plots for ADA with the CDK2 are shown in Figures 6A,B. Molecular docking studies revealed that ADA bound significantly with protein CDK2, with the lowest binding energy of −9 kcal/mol and an inhibitory concentration (Ki) of 21 µM. Also, ADA formed pi-alkyl interaction with Ile10, Leu134, and Ala144, conventional hydrogen bond with Lys19 and Asp145 residues as shown in Figure 6B. Other non-bonded interactions, such as van der Waals interactions involved with Gly11, Glu12, Thr14, Ala31, His84, Gln85, Asp86, Gln131, and Asn132, were also found between CDK2 and ADA. All the binding energy scores are calculated from the best cluster (95%), which falls within the lowest RMSD 0.25 Å. Therefore, from the docking studies, it can be suggested that ADA has a high affinity for CDK2 and was considered for further MD simulation studies.

### Molecular dynamics and simulation

Molecular dynamics and simulation (MD) studies were carried out to determine the stability and convergence of CDK2 with ADA. Simulation of 100 ns displayed stable conformation while comparing the root mean square deviation (RMSD) values. The RMSD of the Cα-backbone of CDK2 bound to ADA exhibited a deviation of 0.5 Å (Figure 6C). Stable RMSD plots during simulation signify a good convergence and stable conformations (Hollingsworth and Dror, 2018). Therefore, it can be suggested that ADA bound to CDK2 is relatively stable in complex due to the higher affinity of the ligand. The plot for root mean square fluctuations (RMSF) displayed small spikes of fluctuation in CDK2 protein except at residues 135 and 146 might be due to the higher flexibility of the residues conformed to the loop region.

In contrast, the rest of the residues fluctuated less during the entire 100 ns simulation, and Figure 6D indicates the stable amino acid conformations during the simulation time. Moreover, all these RMSF values are in the acceptable region. Therefore, from the RMSF plot, it can be suggested that the structure of CDK2 is stable during simulation in ligand-bound conformation (Hollingsworth and Dror, 2018). The radius of gyration (Rg) measures the compactness of the protein. Here, in this study, CDK2 Cα-backbone bound to ADA displayed a stable radius of gyration (Rg) from 19.5 to 19.7 Å (Figure 6E). Significantly, stable gyration (Rg) indicates a highly compact protein orientation in the ligand-bound state (Mosquera-Yuqui et al., 2020). The number of hydrogen bonds between protein and ligand suggests the significant interaction and stability of the complex, and we observed a high number of H-bonds between CDK2 with ADA throughout the simulation time of 100 ns (Figure 6F). Following Rg analysis, similar patterns were observed in a solvent accessible surface area (SASA) in both ligand-bound and ligand-unbound states. In the unbound state of ADA, CDK2 displayed high surface area accessible to solvent (Figure 6G). The SASA value lowered significantly in the bound state with CDK2 as compared to the unbound state. The overall study of Rg signifies that the binding of ADA to CDK2 compels the protein to become more compact and less flexible.

### Adapalene inhibits CDK2 and promotes S-phase cell arrest

In addition, we evaluated the effect of ADA on CDK2 protein levels. It was found that ADA significantly reduced CDK2 protein levels dose-dependent. Moreover, reduced CDK2 protein levels confirm the *in silico* docking and molecular simulation results (Figures 6H,I).

**FIGURE 7:**
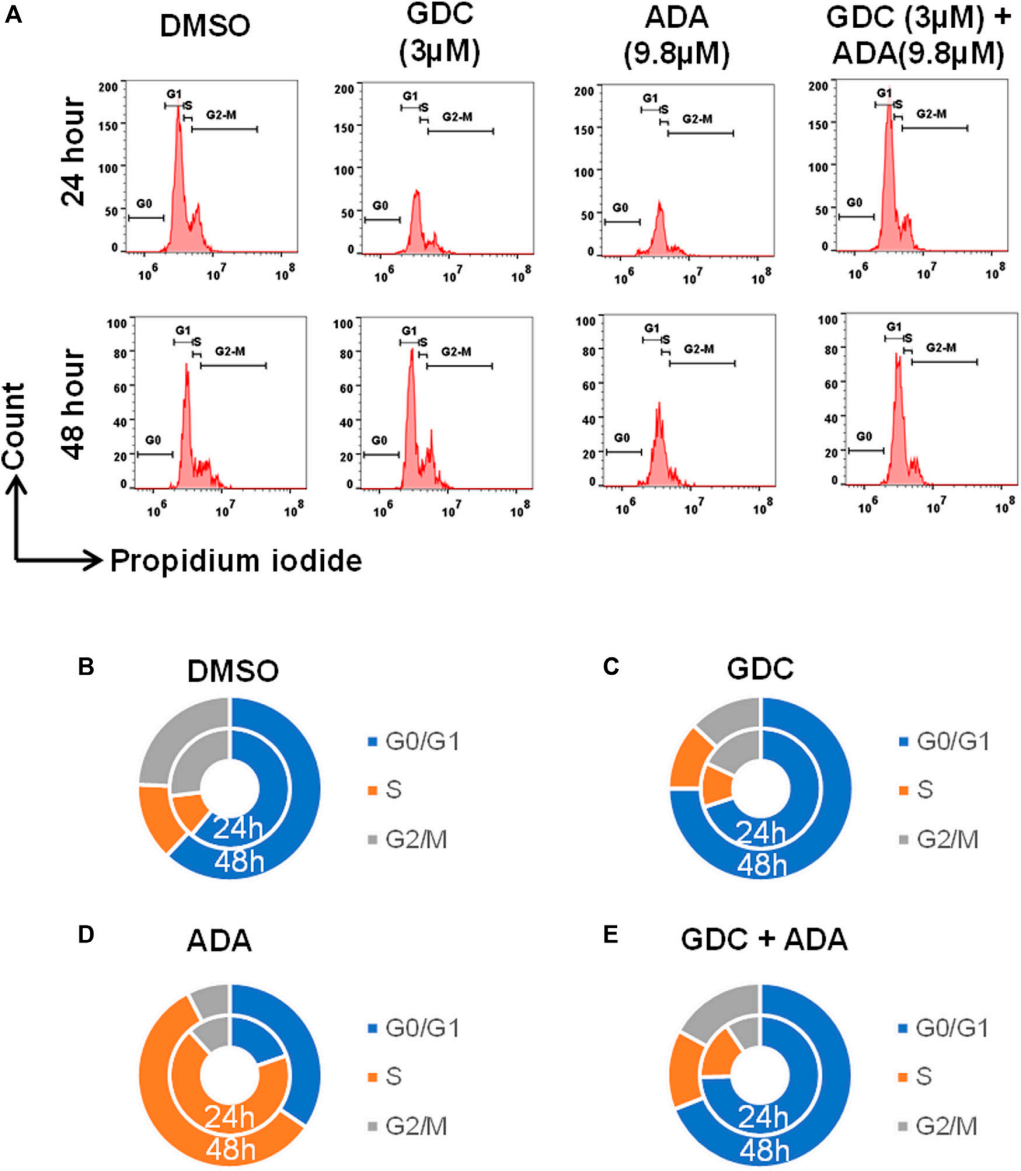
GDC-0941 and adapalene promoted cell cycle arrest of MDA-MB-231 cells **(A–E)** ADA upon treatment showed S-phase arrest of MDA-MB-231 cells, while GDC showed the arrest of MDA-MB-231 cells in the G2/M phase of the cell cycle. Upon combination treatment with GDC and ADA, the arrest of cells enhanced in the S-phase.

We also evaluated the effect of ADA, GDC, and both on the cell cycle. The cell cycle analysis was performed, following the treatment for 24 and 48 h using flow cytometry. Flow cytometry results demonstrated that ADA induced S-phase cell cycle arrest in MDA-MB-231, while GDC promoted the arrest of MDA-MB-231 cells in the G1 phase. In combination, GDC and ADA enhanced the arrest of cells in the G1 phase (Figure 7). Flow cytometry results are in tandem with *in silico* and *in vitro* results, validating that ADA inhibits CDK2, thereby promoting the arrest of cells.

## Discussion

BC is currently one of the most common malignancies and the leading cause of tumor-related mortality in women globally (Siegel et al., 2021; Sung et al., 2021). TNBC is one of the aggressive subtypes of breast cancer, and it has a more aggressive clinical course than the other types of breast cancer (Dai et al., 2016; Mehraj et al., 2022c). Since presently available endocrine and *HER2*-directed medications are inadequate in treating TNBC, chemotherapy has traditionally been the backbone of systemic treatment for the disease (Waks and Winer, 2019; Mehraj et al., 2022d). The PI3K inhibitor, GDC-0941 (Pictilisib), is an orally accessible compound that binds to and competes with the ATP-binding pocket in the PI3K signaling and blocks the signaling cascade (Yamamoto et al., 2017; Han et al., 2019). Patients with advanced NSCLC in Japan reported that it had a superior tolerance and safety profile (Yamamoto et al., 2017). GDC-0941 revealed modest antitumor efficacy in clinical studies, and a tolerable safety profile and combination with paclitaxel improved the antitumor response (Sarker et al., 2015; Schmid et al., 2016). While inhibiting PI3K provides significant therapeutic advantages, there has been some apprehension about the sometimes significant toxicity connected with its usage (Greenwell et al., 2017). As a result, there is an urgent need to investigate innovative therapeutics that will allow for a reduction in the dose of GDC-0941 while simultaneously increasing its antitumor efficacy.

Adapalene, a third-generation retinoid currently used to treat acne, binds to nuclear retinoic acid receptors and has been reported to possess potent antitumor activity (Rusu et al., 2020). ADA possesses high comedolytic, anti-inflammatory, antiproliferative, and immunomodulatory properties. Also, its safety profile is superior to other retinoids (Rusu et al., 2020). Several recent studies established the antitumor potential of ADA in solid malignancies, including HCC, colon cancer, melanoma, ovarian cancer, and prostate cancer (Shi et al., 2015; Ghosalkar et al., 2018; Li et al., 2019; Wang et al., 2019; Nong et al., 2022). Also, in our recent study, we demonstrated the antitumor potential of ADA in TNBC *in vitro* models. We reported that ADA promotes tumor cell apoptosis and acts synergistically with doxorubicin. Herein, we aimed to evaluate the therapeutic potential of ADA in combination with GDC-0941.

Our results demonstrate that ADA is an effective therapeutic agent combined with GDC in inhibiting proliferation, migration, and colonization capacity. We found that the combination of ADA and GDC resulted in a marked increase in cell death in TNBC cancer cells and reduced GDC resistance. ADA enhanced ROS generation and demonstrated a synergistic effect in ROS production and decreased MMP with GDC. Elucidation of the underlying mechanisms warrants further study.

Our findings demonstrate that both ADA alone and in combination with GDC exerts anticancer efficacy by inducing apoptosis in tumor cells. In addition, the synergistic combination of GDC and ADA resulted in considerable dosage reductions for both GDC and ADA, as shown in Tables 1, 2. As shown in Figure 2B, the combination of GDC and ADA showed a synergistic relationship, as depicted by the thickness of the line connecting polygonograms. In addition, it was observed that ADA alone or in combination with GDC inhibited TNBC cell colony formation and migration. Colony numbers and sizes decreased considerably when cells were treated with ADA and GDC in combination.

Cancer cells often produce and sustain larger reactive oxygen species (ROS) levels than normal cells. Increased ROS levels make cancer cells susceptible to ROS-generating agents (Trachootham et al., 2009; Gorrini et al., 2013). As a result, stimulating ROS is a possible therapeutic method for cancer. Numerous studies have shown that increasing ROS production in cancer cells inhibits development and induces apoptosis (Yang et al., 2017; Liu et al., 2018; Baptista Moreno Martin et al., 2020). We found that enhanced ROS levels accumulated upon combined treatment with ADA and GDC and promoted tumor cell apoptosis. Also, in our previous study, we reported that ADA promoted Erk1/2 activation by ROS generation and apoptosis. Previously, Erk1/2 activation has been reported to promote therapeutic resistance, including GDC-0941, how hyperactivation of Erk1/2 by ROS generation prompts apoptotic role of Erk1/2. The previous reports and the present findings indicate that high ROS accumulation upon co-treatment with GDC and ADA reduced GDC resistance *via* modulation of the signaling cascades involved in GDC resistance in TNBC cells. Also, Annexin V/7-AAD labeling revealed that ADA causes apoptosis in MDA-MB-231 cells and that synergistic drug interactions occurred during apoptosis. Apoptosis increased from 24.2 to 40.3 percent in cells treated with the combination of GDC and ADA. These findings suggest that co-treatment with ADA and GDC increases BC cells sensitivity to GDC and that substantial tumor inhibition may be obtained with moderate dosages of both drugs, hence decreasing the risks associated with GDC-0941.

In addition, it was further found that ADA selectively binds CDK2 and induces S-phase cell cycle arrest. The results were validated both *in silico* and *in vitro*. CDK2 is upregulated in breast tumors, and its vital role in cell cycle regulation makes CDK2 an attractive therapeutic target (Sofi et al., 2022a; Mehraj et al., 2022e; Mehraj et al., 2022f). Together, these results demonstrate that ADA has high potential in reducing tumor growth and colonization of TNBC cells. A recent study demonstrated that ADA-mediated tumor growth suppression occurs due to DNA damage and apoptotic pathway activation (Nong et al., 2022). Also, Wang et al. (2019) demonstrated that ADA reduced the growth of ovarian cancer cells by inhibiting glutamic-oxaloacetic transaminase 1. ADA also effectively reduced the growth of colorectal carcinoma, melanoma cells, prostate cancer cells, and hepatoma cells (Ocker et al., 2004; Shi et al., 2015; Li et al., 2019; Nong et al., 2022). These previous studies and the present study’s findings demonstrate that ADA is a promising therapeutic agent across a wide range of tumors with TNBC, prostate tumors, and melanoma cells more responsive toward ADA treatment.

In addition to its enhanced ability to inhibit proliferation and promote tumor cell apoptosis, ADA may offer numerous advantages over conventional retinoic acid derivatives *in vivo*, such as increased stability, more prolonged comedolytic action, enhanced anti-inflammatory activity, and a favorable safety profile (Ocker et al., 2003; Shi et al., 2015; Ghosalkar et al., 2018; Rusu et al., 2020; Sofi et al., 2022b).

In conclusion, our results indicate that the combination of ADA and GDC is effective against BC cells by augmenting the induction of apoptosis. Existing chemotherapy agents are associated with highly unpleasant side effects, but ADA has the potential to be an effective anticancer treatment with far reduced toxicity. Even though our results were verified *in vitro*, they paved the way for further exploration of the synergistic therapeutic combination of GDC and ADA *in vivo* and with other TNBC models and cancer hallmarks.

## Data Availability

The raw data supporting the conclusion of this article will be made available by the authors, without undue reservation.
