# Supplementary material for: Adapalene inhibits the growth of triple-negative breast cancer cells by S-phase arrest and potentiates the antitumor efficacy of GDC-0941
Source: Front Pharmacol. 2022 Aug 8;13:958443. doi: 10.3389/fphar.2022.958443 (PMC9393306; doi:10.3389/fphar.2022.958443)
Supplement: Supplementary file 1 [file DataSheet1.PDF]

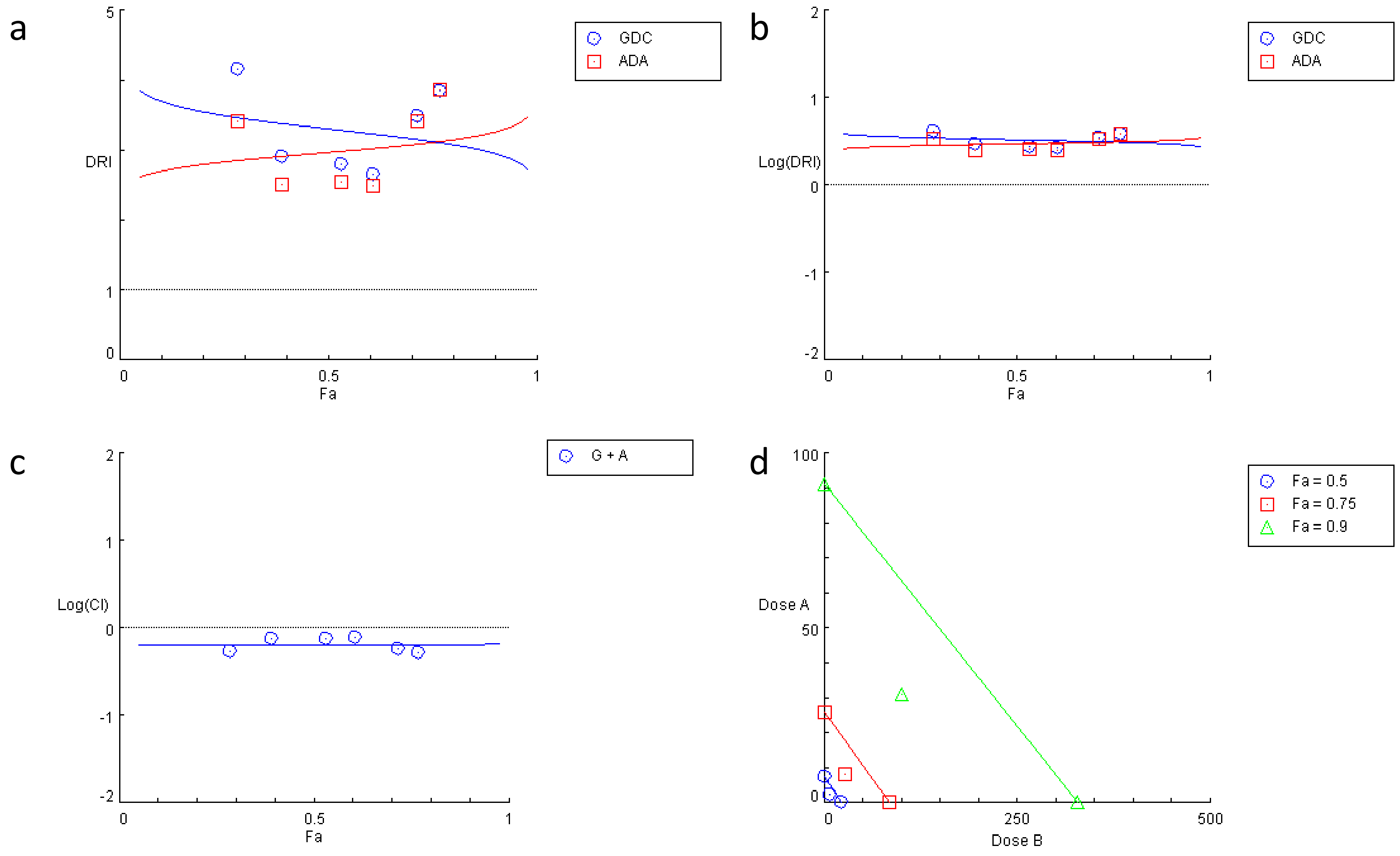

Figure S1. CompuSyn report of MDA-MB-231: a. DRI Plot b. Log DRI, c. Log(CI), d. Isobologram

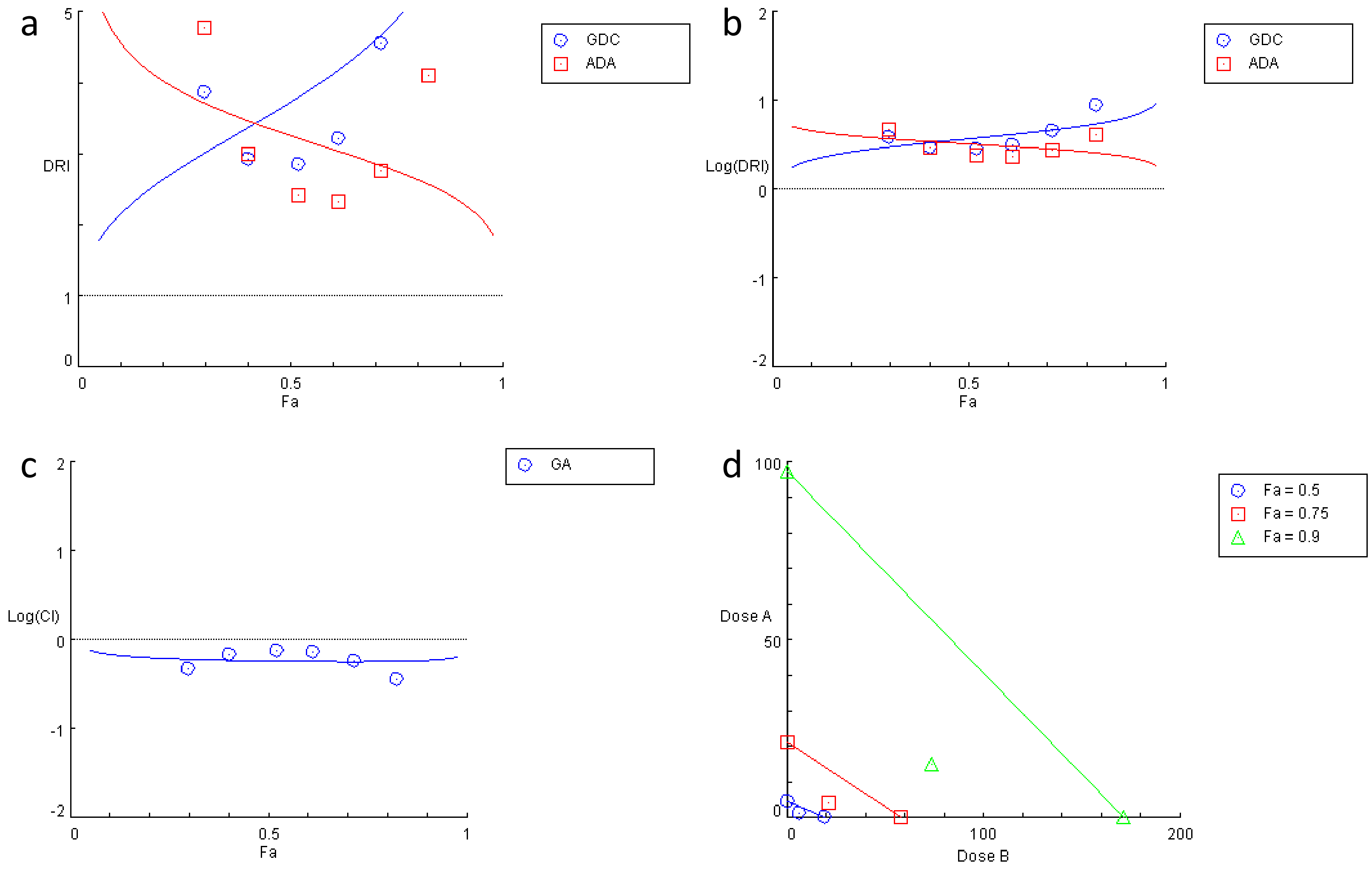

Figure S2. CompuSyn report of MDA-MB-468: a. DRI Plot b. Log DRI, c. Log(CI), d. Isobologram

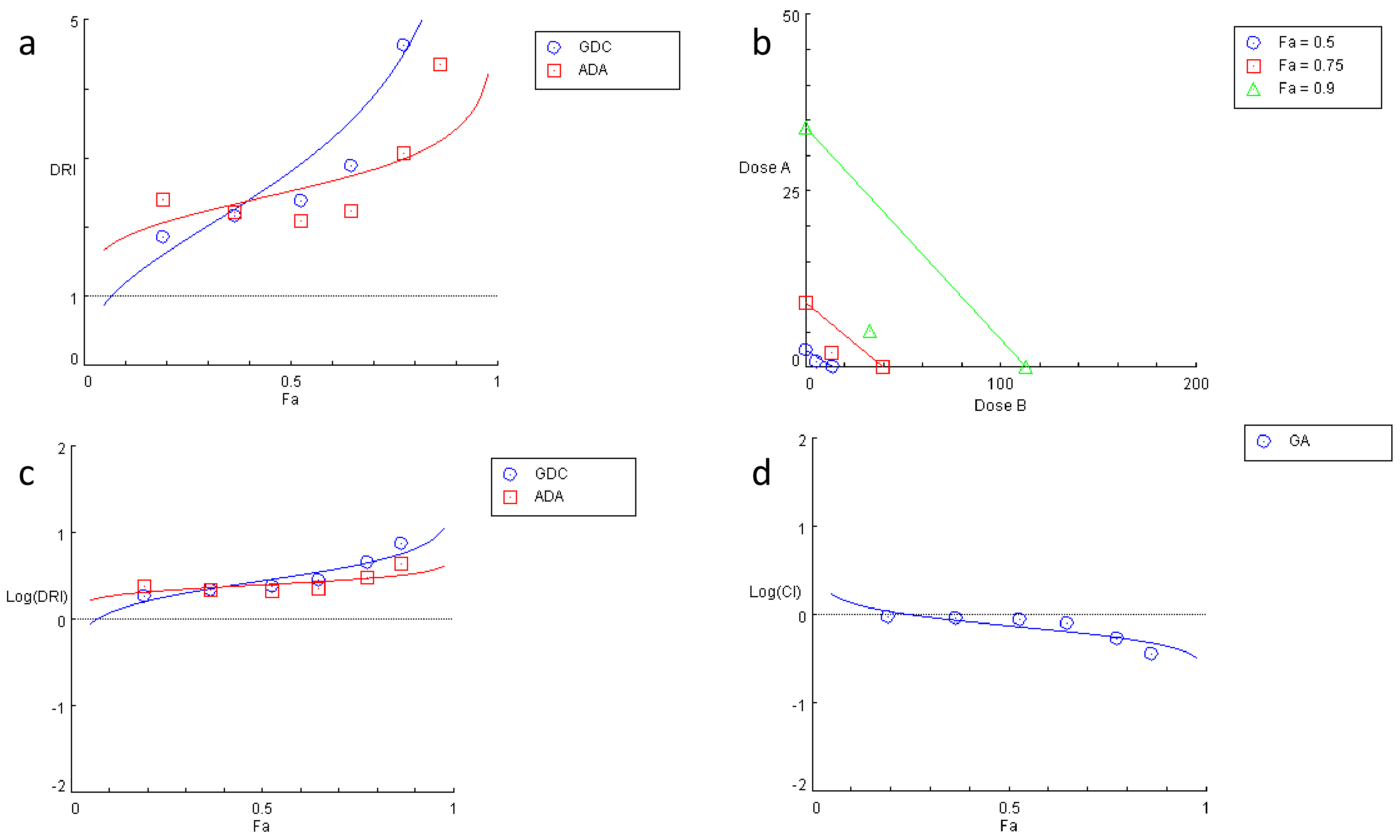

Figure S3. CompuSyn report of 4T1: a. DRI Plot b. Isobologram, c. Log DRI, d. Log(CI).

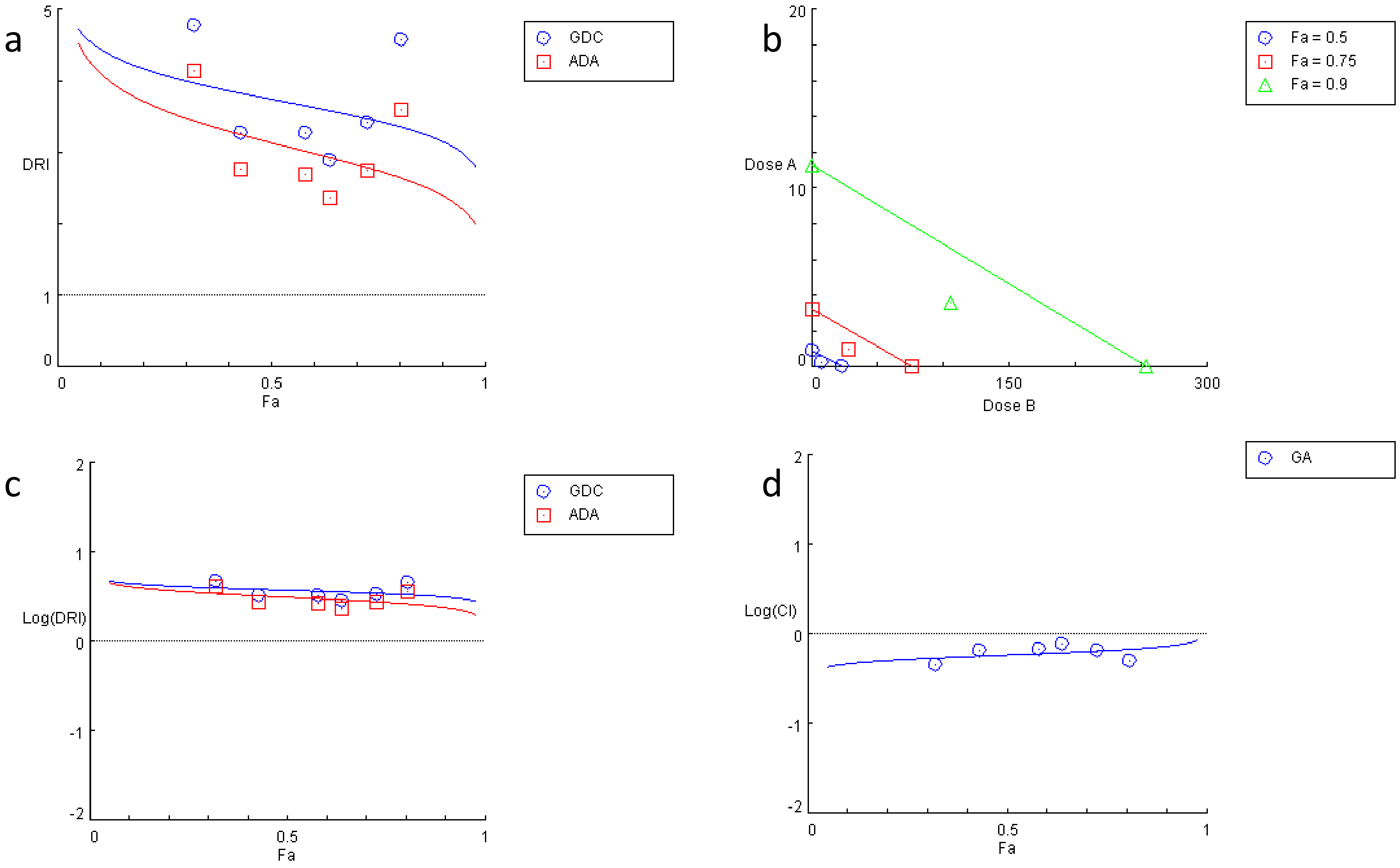

Figure S4. CompuSyn report of MCF-7: a. DRI Plot b. Isobologram, c. Log DRI, d. Log(CI)
